# Diagnostic Performance of a Combined Rapid Antigen Test for Detecting SARS‐CoV‐2, Influenza Virus, and Respiratory Syncytial Virus in Symptomatic Patients in Tertiary Care

**DOI:** 10.1002/jmv.70493

**Published:** 2025-07-16

**Authors:** Jakob Meyer, Rainer Gosert, Roland Bingisser, Christian H. Nickel, Sarah Tschudin‐Sutter, Karoline Leuzinger

**Affiliations:** ^1^ Emergency Medicine University Hospital Basel Basel Switzerland; ^2^ Clinical Virology University Hospital Basel Basel Switzerland; ^3^ Infectious Diseases University Hospital Basel Basel Switzerland; ^4^ Department of Clinical Research University Hospital Basel Basel Switzerland

**Keywords:** influenza, influenza virus, rapid antigen test, rapid diagnostic test, RAT, RDT, respiratory syncytial virus, respiratory tract infection, RSV, SARS‐CoV‐2

## Abstract

Rapid antigen diagnostic tests (RDTs) can rapidly detect respiratory pathogens, allowing for the prompt initiation of infection control measures and the prevention of nosocomial spread within hospital settings. In this study, we prospectively evaluated the diagnostic performance of a combined RDT from AllTest Biotech for the simultaneous detection of SARS‐CoV‐2, influenza virus (IV‐A/B), and respiratory syncytial virus (RSV). We compared its diagnostic performance to the Xpert‐Xpress‐SARS‐CoV‐2/Flu/RSV molecular test using 100 naso‐oropharyngeal swabs (Ct‐values ≤ 35), collected from symptomatic patients with acute respiratory tract infections (RTIs) at our tertiary care hospital. The RDT showed a sensitivity of 60% (95%CI: 43.4%–74.7%) for SARS‐CoV‐2, with lower sensitivities for RSV at 60.0% (95%CI: 38.9%–78.2%) and IV‐A/B at 54.3% (95%CI: 36.9%–70.8%). Higher sensitivities of 100% were achieved for all three viruses in respiratory samples with higher viral loads (Ct‐values ≤ 25). The RDT demonstrated high specificity of > 99% for SARS‐CoV‐2, IV‐A/B, and RSV. In conclusion, the Alltest‐SARS‐CoV‐2/IV‐A + B/RSV RDT is effective for detecting SARS‐CoV‐2, IV‐A/B, and RSV in samples with high viral loads, but its sensitivity significantly declines at Ct‐values above 25. Therefore, negative RDT results should be confirmed with nucleic acid testing in symptomatic patients with RTIs to prevent severe consequences for clinical management.

## 1 Introduction

Rapid detection of SARS‐CoV‐2, influenza virus A and B (IV‐A/B), and respiratory syncytial virus (RSV), causing acute respiratory tract infections (RTIs) with overlapping clinical symptoms, is essential in health care settings to implement infection control measures and guide clinical management. Nucleic acid testing (NAT) remains the gold standard due to its high sensitivity but requires specialized infrastructure and is typically associated with longer turnaround times. In contrast, rapid antigen diagnostic tests (RDTs) offer simpler workflows, faster results (within 10–15 min), and lower per‐test costs. These features make RDTs well‐suited for tertiary care settings, where rapid diagnosis is critical for patient triage and timely admission to inpatient care. However, ensuring the diagnostic reliability of RDTs outside centralized laboratory settings requires structured workflows, robust documentation with automated result reporting, properly trained personnel, and strict adherence to quality assurance protocols. In previous studies, we reported good RDT performance for SARS‐CoV‐2 detection in patients with high viral loads (Ct‐value ≤ 25), with sensitivities of 91%–98% [1, 2]. Given the seasonal co‐circulation of SARS‐CoV‐2, IV‐A/B, and RSV, combined RDTs have gained relevance in clinical practice. A recent study evaluating three combined SARS‐CoV‐2/Flu/RSV RDTs demonstrated their potential to support rapid identification of co‐circulating respiratory viruses in clinical settings [3]. However, prior research has shown that RDT sensitivity can be affected by several factors, including variation in the viral antigens targeted by RDTs and patient‐related characteristics such as age, immune status, and vaccination history [4, 5]. Understanding these factors is essential in tertiary care settings to ensure diagnostic accuracy, support clinical decision‐making, and guide effective patient management.

In this prospective study, we assessed the diagnostic performance of a combined SARS‐CoV‐2, IV‐A/B and RSV RDT in comparison to the Xpert‐Xpress‐SARS‐CoV‐2/Flu/RSV using 100 respiratory swabs from symptomatic patients presenting with RTIs at our tertiary care hospital.

## 2 Materials and Methods

### 2.1 Clinical Specimens and NAT Testing

Naso‐oropharyngeal swabs were collected from symptomatic patients presenting with acute RTIs, defined by the presence of at least one respiratory symptom (e.g., nasal congestion, rhinorrhea, sore throat, or cough) and one systemic symptom (e.g., fever, fatigue, headache, chills, or myalgia), in accordance with established criteria [6]. Swabs were placed in universal transport medium (UTM; Copan, Brescia, Italy), and immediately analyzed using the Xpert‐Xpress‐SARS‐CoV‐2/Flu/RSV plus test (Cepheid, CA, USA) at the Clinical Virology unit, University Hospital Basel (UHB), Switzerland, as previously described [7]. Ct‐values were automatically generated by the Xpert‐Xpress‐SARS‐CoV‐2/Flu/RSV for each pathogen based on predefined multi‐gene targets. For IV‐A, the reported Ct‐values correspond to the matrix gene target. These values were used as semi‐quantitative measures of viral RNA levels but were not calibrated to absolute viral loads.

### 2.2 Combined SARS‐CoV‐2, IV‐A/B and RSV RDT

From March to June 2024, 100 consecutive symptomatic patients were enrolled in the study cohort; no asymptomatic or presymptomatic patients were included. The diagnostic performance of the SARS‐CoV‐2/IV‐A + B/RSV Antigen Combo Rapid Test (AllTest Biotech, Hangzhou, China) was assessed using 50 SARS‐CoV‐2–positive, 25 influenza A/B–positive, and 25 RSV‐positive samples, all with Ct‐values ≤ 35 on the Xpert‐Xpress‐SARS‐CoV‐2/Flu/RSV. The AllTest‐SARS‐CoV‐2/IV‐A + B/RSV is CE‐marked and conforms to EU IVD regulatory requirements. RDTs were performed according to the manufacturer's instructions within 24 h of sample collection, using naso‐oropharyngeal swabs stored in UTM at 4°C. Test results were read after 10 min. Readings were performed independently by two laboratory technicians, blinded to the Xpert‐Xpress‐SARS‐CoV‐2/Flu/RSV results. All RDTs yielded valid results, with no invalid or indeterminate results.

### 2.3 Statistical Methods

Sensitivity and specificity of the RDT was calculated according to the results of the Xpert‐Xpress‐SARS‐CoV‐2/Flu/RSV. Receiver operating characteristic analysis was done using the sensitivities of each RDT stratified by viral load. All statistical data analysis was done in R (version 4.3.3; https://cran.r-project.org), using Prism (version 10; Graphpad Software, CA, USA) for data visualization. Mann–Whitney‐U test was used as indicated.

## 3 Results

### 3.1 Detection of Respiratory Viruses by the Xpert‐Xpress‐SARS‐CoV‐2/Flu/RSV

**Figure 1:**
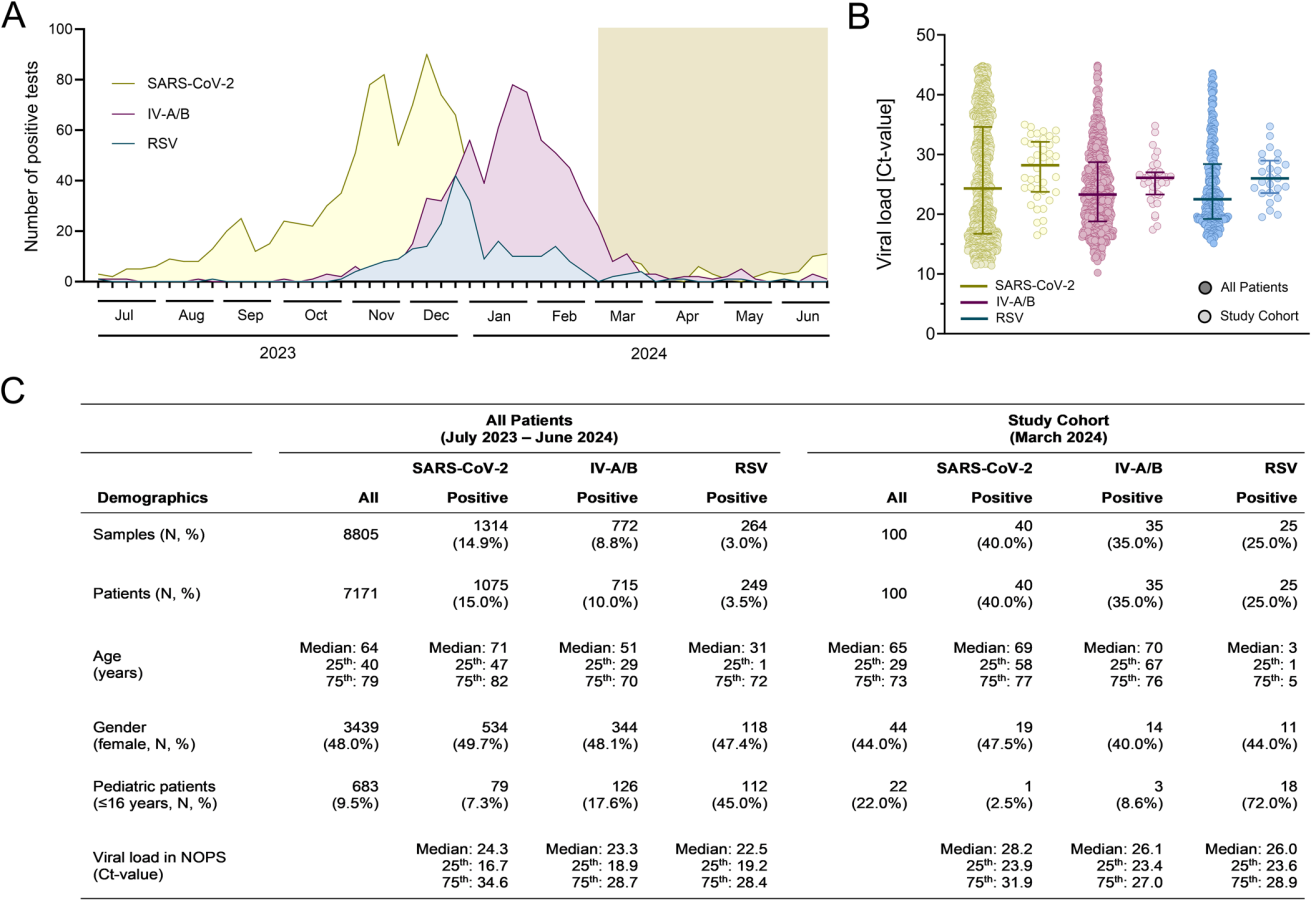
Seasonal patterns and demographic characteristics of patients with SARS‐CoV‐2, influenza virus A/B and respiratory syncytial virus infections. (A) Weekly number of SARS‐CoV‐2, influenza virus A/B (IV‐A/B), and respiratory syncytial virus (RSV) detections using the Xpert Xpress SARS‐CoV‐2/Flu/RSV assay at our center from July 2023 to June 2024. The study period from March to June 2024 is highlighted with a gold‐colored box. (B) Initial respiratory virus loads (cycle threshold values) in symptomatic patients at the time of hospital presentation, shown for both the full patient population tested by the Xpert Xpress SARS‐CoV‐2/Flu/RSV assay (*n* = 2039) and the subset included in the RDT evaluation study (*n* = 100). (C) Patients' demographics from all patients tested by the Xpert Xpress SARS‐CoV‐2/Flu/RSV assay and the subset included in the RDT evaluation study.

From July 2023 to June 2024, detection rates for RSV and IV‐A/B were seasonal, with peaks in December and January, respectively, while SARS‐CoV‐2 activity peaked in December but remained detectable year‐round with a positivity rate ranging from 5% to 10% (Figure 1A). Upon hospital presentation, patients showed median Ct‐values of 24 for SARS‐CoV‐2 (range: 11–44), 23 for IV‐A/B (range: 10–44), and 23 for RSV (range: 15–44; Figure 1B). Respiratory swabs were submitted from 6488 (90.5%) symptomatic adult and 683 (9.5%) pediatric patients (Figure 1C). The median age of SARS‐CoV‐2–positive patients was 71 years, while IV‐A/B– and RSV–positive patients were significantly younger at 51 and 31 years (*p* < 0.001; Figure 1C). The study cohort used to evaluate RDT performance comprised a subset of 100 patients from this overall population.

### 3.2 Comparison of Respiratory Virus Detection Between the Xpert‐Xpress‐SARS‐CoV‐2/Flu/RSV and the Alltest‐SARS‐CoV‐2/IV‐A + B/RSV RDT

**Figure 2:**
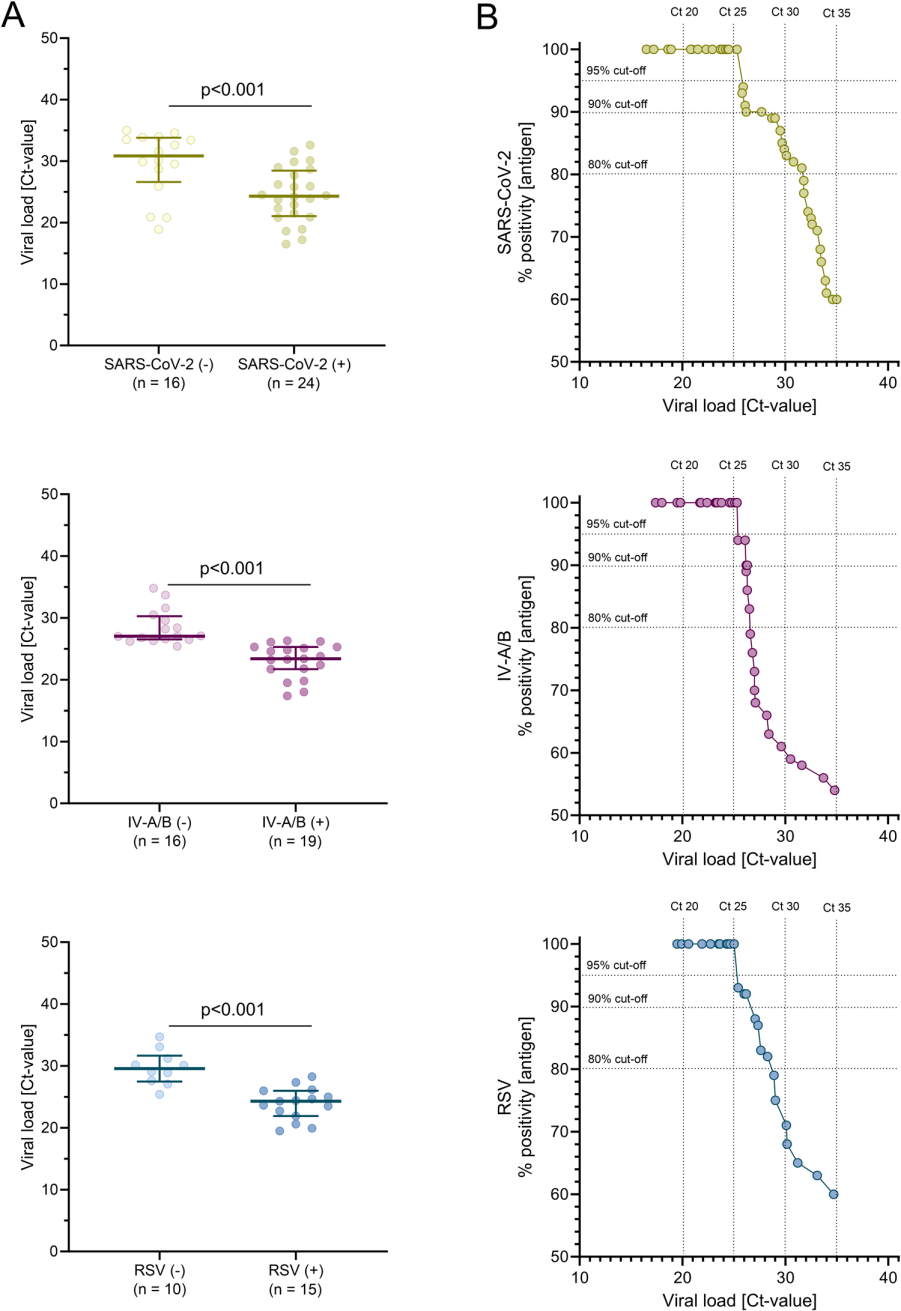
SARS‐CoV‐2, influenza virus A/B and respiratory syncytial virus detection with the Alltest‐SARS‐CoV‐2/IV‐A + B/RSV. (A) Respiratory virus load (cycle threshold value) in samples with positive and negative results by the Alltest‐SARS‐CoV‐2/IV‐A + B/RSV rapid antigen test (*n* = 100; median, 25th and 75th percentile; Mann‐Whitney‐U test). (B) Cumulated sensitivity of the Alltest‐SARS‐CoV‐2/IV‐A + B/RSV based on respiratory virus load (*n* = 100).

We prospectively evaluated 100 consecutive naso‐oropharyngeal swab samples and compared the results to the Xpert‐Xpress‐SARS‐CoV‐2/Flu/RSV. All virus–negative samples were nonreactive in the Alltest‐SARS‐CoV‐2/IV‐A + B/RSV, yielding a specificity of 100% for SARS‐CoV‐2, IV‐A/B, and RSV. Among the 40 SARS‐CoV‐2–positive samples, 24 were correctly detected by the RDT, corresponding to an overall agreement of 84% and a moderate Cohen's kappa coefficient of 0.64 (Supporting Information Table 1A). For IV‐A/B, the Alltest‐SARS‐CoV‐2/IV‐A + B/RSV identified 19 of the 25 IV‐A/B–positive samples, corresponded to an overall agreement of 84% and a moderate Cohen's kappa coefficient of 0.61 (Supporting Information Table 1B). For RSV, the Alltest‐SARS‐CoV‐2/IV‐A + B/RSV detected 15 of the 25 RSV–positive samples, resulting in an overall good agreement of 90%, and a Cohen's kappa coefficient of 0.69 (Supporting Information Table 1C). However, discrepancy rates between 40% and 46% were observed across all three respiratory viruses when using the Alltest‐SARS‐CoV‐2/IV‐A + B/RSV (Supporting Information Table 1A–C). Comparison of viral loads between concordant and discordant samples revealed significantly lower Ct‐values in concordant positives than in discordant cases (*p* < 0.001; Figure 2A). These findings suggest that the discordant results likely occurred near the detection limit of the Alltest‐SARS‐CoV‐2/IV‐A + B/RSV.

### 3.3 Diagnostic Performance of the Alltest‐SARS‐CoV‐2/Iv‐A + B/RSV RDT Based on Respiratory Virus Load

To further evaluate the diagnostic performance of the Alltest‐SARS‐CoV‐2/IV‐A + B/RSV, RDT sensitivity was assessed in relation to respiratory virus load. Across the full data set of all available samples with Ct‐values ≤ 35, overall RDT sensitivity was 60.0% (95%CI: 43.4%–74.7%) for SARS‐CoV‐2, 54.3% (95%CI: 36.9%–70.8%) for IV‐A/B, and 60.0% (95%CI: 38.9%–78.2%) for RSV. In samples with Ct‐values ≤ 30, sensitivity improved to 81.4% (95%CI: 61.3%–93.0%) for SARS‐CoV‐2, 59.4% (95%CI: 40.8%–75.8%) for IV‐A/B, and 68.2% (95%CI: 45.1%–85.3%) for RSV (Table 1, Figure 2B). Sensitivity increased further with higher viral loads, reaching 100% for all three viruses in samples with Ct‐values ≤ 25 (Figure 2B).

**Table 1:** Alltest‐SARS‐CoV‐2/IV‐A + B/RSV test characteristics for SARS‐CoV‐2, influenza virus A/B, and respiratory syncytial virus detection.

Respiratory viral pathogen^a^	SARS‐CoV‐2					Influenza virus A/B					Respiratory syncytial virus				
Virus detection rate	1%	5%	10%	15%	20%	1%	5%	10%	15%	20%	1%	5%	10%	15%	20%
Sensitivity [%, 95% CI]	81.4 (61.3–93.0)					59.4 (40.8–75.8)					68.2 (45.1–85.3)				
Specificity [%, 95% CI]	100 (92.5–100)					100 (93.0–100)					100 (93.9–100)				
False positive rate [%]	0 (0–18.5)					0 (0–20.9)					0 (0–25.3)				
False negative rate [%]	7.7 (2.9–17.8)					16.7 (9.5–27.2)					8.5 (3.8–17.3)				
Positive predictive value [%, 95% CI]	100 (84.6–100)					100 (82.4–100)					100 (78.2–100)				
Negative predictive value [%, 95% CI]	99.8 (99.6–100)	99.0 (97.9–99.6)	98.0 (95.7–99.1)	96.8 (93.3–98.5)	95.6 (90.7–97.9)	99.6 (99.4–99.7)	97.9 (96.9–98.6)	95.7 (93.6–97.1)	93.3 (90.2–95.5)	90.8 (86.6–93.7)	99.7 (99.4–99.8)	98.4 (97.0–99.1)	96.6 (93.9–98.1)	94.7 (90.6–97.0)	92.6 (87.2–95.9)
Accuracy [%, 95% CI]	99.8 (95.5–100)	99.1 (94.1–100)	98.2 (92.6–99.9)	97.2 (91.2–99.6)	96.3 (89.9–99.2)	99.6 (95.5–100)	98.0 (92.8–99.8)	95.9 (89.9–98.9)	93.9 (87.1–97.8)	91.9 (84.6–96.5)	99.7 (95.7–100)	98.4 (93.5–99.9)	96.8 (91.1–99.3)	95.2 (88.9–98.5)	93.6 (86.8–97.6)

^a^ Including virus RNA positive samples with Xpress SARS‐CoV‐2/Flu/RSV Ct‐values < 30, SARS‐CoV‐2 (*n* = 87), influenza virus A and B (*n* = 97), respiratory syncytial virus (*n* = 97).

**Figure 3:**
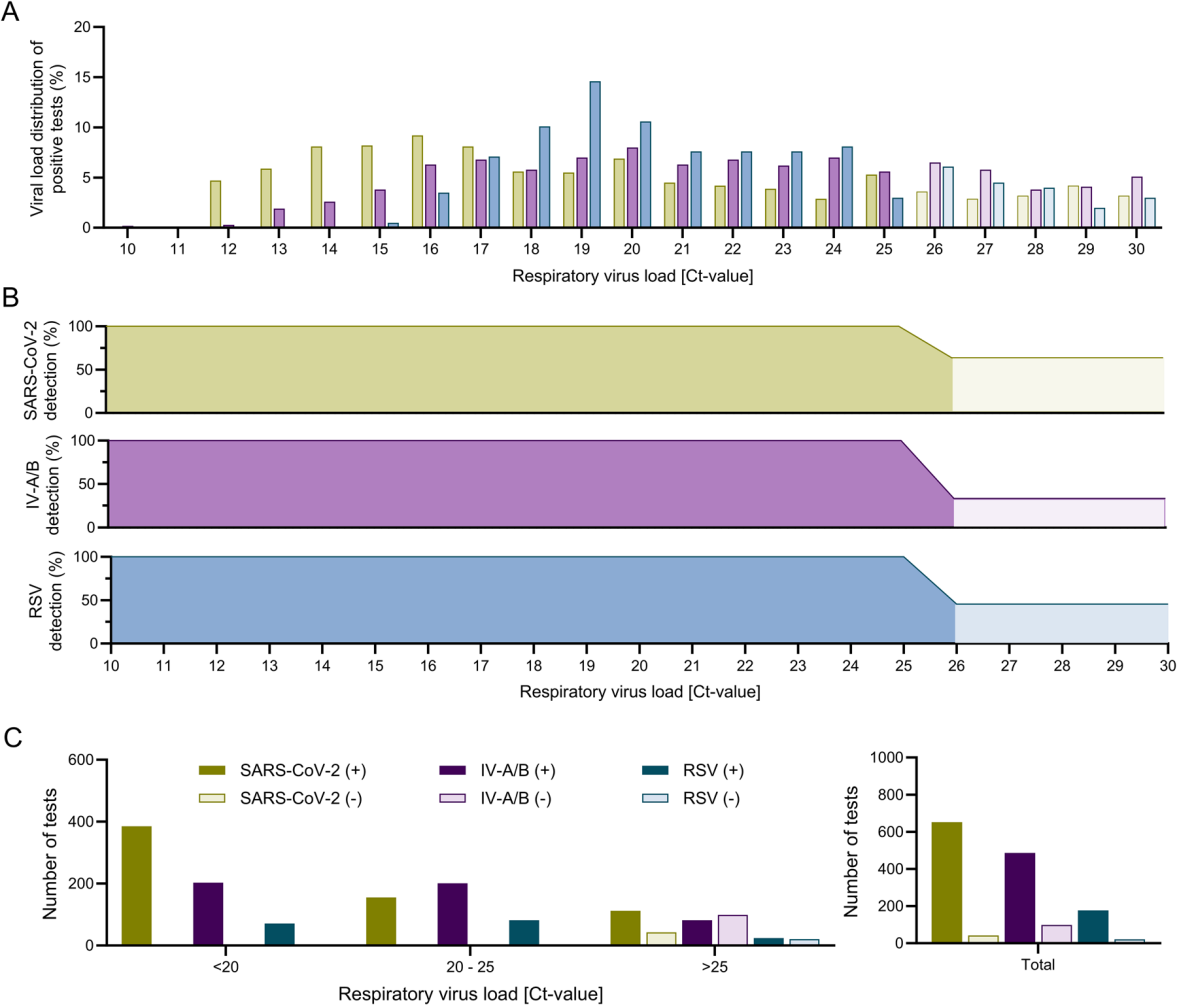
Diagnostic performance of the Alltest‐SARS‐CoV‐2/IV‐A + B/RSV. (A) Viral load distribution (cycle threshold (Ct) values) as determined by the Xpert Xpress SARS‐CoV‐2/Flu/RSV test for SARS‐CoV‐2, IV‐A/B and RSV from July 2023 to June 2024 (*n* = 2350 total viral detections). (B) Sensitivity of the Alltest‐SARS‐CoV‐2/IV‐A + B/RSV for detecting SARS‐CoV‐2, IV‐A/B and RSV based on viral load. (C) Number of detected positive cases based on viral loads.

When assessing viral loads measured by the Xpert‐Xpress‐SARS‐CoV‐2/Flu/RSV from June 2023 to July 2024, we found that 1478 out of 2039 (72.5%) samples had Ct‐values ≤ 30 (Figure 3A). When applying sensitivity thresholds based on viral loads, the Alltest‐SARS‐CoV‐2/IV‐A + B/RSV would have detected 652 of 695 (93.8%) SARS‐CoV‐2–positive cases, 486 of 585 (83.1%) IV‐A/B–positive cases, and 177 of 198 (89.3%) RSV–positive cases with Ct‐values ≤ 30 (Figure 3B,C).

## 4 Discussion

Rapid detection of SARS‐CoV‐2, IV‐A/B, and RSV is critical for informed clinical decision‐making in tertiary care. It enables timely implementation of isolation protocols and infection control measures to reduce the risk of nosocomial transmission. In addition, it supports efficient resource management by guiding the allocation of ICU beds, medical staff, and treatments to the patients who need them most. NAT enables rapid and highly sensitive detection of respiratory viruses from clinical specimens, often delivering results in under an hour [8, 9, 10]. However, NAT requires specialized equipment, trained personnel, and is associated with higher per‐test costs. RDTs provide results within 10–15 min, require minimal laboratory infrastructure, and have lower operational costs, making them suitable for broader implementation across various healthcare settings. Still, the accuracy of RDTs depends on well‐trained personnel, and strict adherence to quality assurance protocols. Furthermore, RDTs must be supported by structured workflows, robust documentation practices, and seamless integration with laboratory information systems to maintain traceability and ensure timely clinical access to test results.

Our study evaluated the Alltest‐SARS‐CoV‐2/IV‐A + B/RSV, a combined RDT for detecting SARS‐CoV‐2, IV‐A/B, and RSV. The test demonstrated high specificity ( ≥ 99%) for all three viruses, consistent with previous reports on RDT performance [1, 2, 11, 12]. Sensitivities were 60.0% for SARS‐CoV‐2, 54.3% for IV‐A/B, and 60.0% for RSV, aligning with published sensitivity ranges [1, 2, 3, 11, 12, 13, 14]. Our findings also confirm that RDT sensitivity is highest in samples with high viral loads (Ct‐value ≤ 25), consistent with prior reports showing reduced sensitivity at Ct‐values above this threshold [1, 2, 3, 13]. Our findings also align with a recent large‐scale evaluation of three multiplex SARS‐CoV‐2/Flu/RSV RDTs in over 1600 symptomatic patients at a tertiary care clinic. Sensitivities exceeded 80% and specificities were generally > 97% for SARS‐CoV‐2, IV‐A, and RSV detection, though one RDT showed reduced specificity for IV‐A [3]. These findings support the potential of combined RDTs for rapid detection of co‐circulating respiratory viruses while underscoring the need for independent validation in clinical settings.

It is important to note that in our study, RDTs were performed under optimal laboratory conditions with trained personnel, high‐quality swabs in UTM, and clear‐positive samples with Ct‐values ≤ 35 on the Xpert‐Xpress‐SARS‐CoV‐2/Flu/RSV. Thus, the reported sensitivity and specificity reflect diagnostic performance under best‐case conditions in terms of sample quality and test execution, and may not fully represent test performance in routine clinical practice, point‐of‐care settings, or unsupervised self‐testing at home, especially with self‐collected respiratory samples. Recent studies have shown that nasopharyngeal and nasal self‐swabbing provide more reliable diagnostic results, whereas buccal swabs tend to yield lower viral RNA levels, especially following food intake [15, 16]. Therefore, sample quality and operational factors are critical to RDT reliability, highlighting the need for robust quality assurance protocols and ongoing staff training.

Furthermore, patient characteristics also influence RDT performance. Our study included only symptomatic individuals, aligning with the intended clinical use of RDTs in emergency and outpatient triage settings. Since sensitivity is significantly lower in asymptomatic or presymptomatic individuals [17], our findings may not be fully generalizable to broader screening contexts. Immune status also significantly influences test performance, with reported sensitivities as low as 32% among vaccinated individuals. Patient age is another important factor affecting test reliability, with RSV sensitivity shown to decrease from 52.3% in infants to 33.3% in children > 5 years [5, 13]. In our study, 80% of RSV–positive samples were from children < 5 years, likely contributing to the observed sensitivity of the RDT.

Finally, the timing of diagnostic testing is also critical. At our tertiary care center, symptomatic patients presented with a wide range of viral loads. RDT performance is typically highest in the first days after symptom onset, when viral RNA levels peak [9, 10, 14, 18], with reported sensitivity ranges from 35.7% to 71.4%, increasing to 90.6% by day four [19]. Although viral detection beyond 10 days after symptom onset is generally not considered to represent infectious virions [20], some samples with Ct‐values up to 35 yielded culturable virus [18]. This indicates that patients with lower viral loads, and corresponding negative RDT results, may still be infectious, especially in the early stages of infection. In contrast, NATs may remain positive for weeks after infection, detecting residual RNA even when infectious virus is no longer present [14]. Notably, immunocompromised individuals and transplant recipients, may continue to shed infectious virus at low viral loads for extended periods [20]. These findings underscore that while high viral loads typically correlate with the presence of culturable virus, neither NAT nor antigen‐based RDTs can reliably distinguish between infectious virus and noninfectious viral remnants. Only virus isolation in cell culture can confirm infectivity, which is not feasible in routine diagnostics. Thus, the relationship between viral load and contagiousness is complex and modulated by host‐ and virus‐specific factors such as immune status, timing of sample collection, and viral replication kinetics. Consequently, both NAT and RDT results must be interpreted with caution and in clinical context, when guiding infection control measures and patient management.

These considerations are particularly relevant when evaluating the clinical utility of RDTs in healthcare settings. Although the evaluated RDT meets the World Health Organization's performance criteria ( ≥ 80% sensitivity and ≥ 97% specificity), its clinical applicability remains limited. While the high specificity observed in our study supports accurate identification of uninfected individuals, even highly specific RDTs may produce a high proportion of false positives during periods of low respiratory virus circulation. Therefore, their use should be guided by local epidemiological trends and supported by confirmatory molecular testing when clinical suspicion is low or testing occurs outside peak virus seasons. Additionally, the moderate sensitivity observed for SARS‐CoV‐2, IV‐A/B, and RSV reduces the reliability of RDTs for comprehensive respiratory virus screening. Collectively, these findings indicate that the clinical utility of RDTs remains limited in high‐risk settings such as tertiary care facilities.

Some limitations should be considered when interpreting the findings of this study. First, we did not include patients who tested negative for all three respiratory viruses. Although this limited the ability to fully assess specificity in a virus‐negative population, our use of mono‐positive samples allowed for the practical evaluation of cross‐reactivity. All samples negative for a given virus, tested negative in the corresponding RDT lane, supporting the test's high analytical specificity. Second, our sample size (*n* = 100) was relatively small, limiting the statistical power for subgroup analyzes. However, the inclusion of well‐characterized, symptomatic patients with a range of viral loads enabled the identification of meaningful trends in diagnostic performance. These results provide a foundation for future studies involving larger and more diverse cohorts to further evaluate the performance of multiplex RDTs. Third, Ct‐values were used as a surrogate marker for viral antigen levels. Although multiple studies have shown a strong overall correlation between Ct‐values and antigen concentrations, this relationship can vary based on factors such as symptom onset, stage of infection, and individual immune response. As such, Ct‐values should be interpreted with caution and not assumed to directly reflect infectiousness or antigen detectability in every case [4]. Fourth, Ct‐values are semi‐quantitative surrogates rather than precise measures of viral burden and are not standardized across NAT platforms. They can vary depending on assay design, chemistry, and amplification conditions. In our study, Ct values were derived from the widely used Xpert‐Xpress SARS‐CoV‐2/Flu/RSV assay, a highly standardized, closed platform that allows for practical comparability across laboratories. Although not calibrated to absolute copy numbers, its reproducibility supports consistent performance. Future studies should incorporate calibrated reference standards to better correlate Ct‐values with viral load and RDT performance.

In summary, the Alltest‐SARS‐CoV‐2/IV‐A + B/RSV RDT is effective in detecting SARS‐CoV‐2, IV‐A/B, and RSV in samples with high viral loads, but is markedly reduced at Ct‐values above 25. Although RDTs offer some technical advantages, their overall moderate diagnostic performance requires cautious interpretation. Future advancements in RDT technology that enhance sensitivity without compromising specificity are needed to broaden their clinical applicability. Until such advancements are achieved, negative RDT results should not be used to rule out infection and must be confirmed by NAT in high‐risk patients or critical care settings to ensure diagnostic accuracy.

## Author Contributions

Jakob Meyer, Rainer Gosert, and Karoline Leuzinger contributed to the conceptualization and study design. Data collection and clinical contributions were provided by Jakob Meyer, Rainer Gosert, Roland Bingisser, Christian H. Nickel, Sarah Tschudin‐Sutter, and Karoline Leuzinger. Methodology and laboratory analyzes were conducted by Rainer Gosert and Karoline Leuzinger. Data analysis and interpretation were performed by Jakob Meyer, Rainer Gosert, and Karoline Leuzinger. The original draft of the article was written by Jakob Meyer, Rainer Gosert, and Karoline Leuzinger. All authors reviewed and approved the final article.

## Ethics Statement

The study was conducted according to good laboratory practice and in accordance with the Declaration of Helsinki and national and institutional standards for laboratory quality control and was approved by the Ethical Committee of North‐Western and Central Switzerland (EKNZ 2020‐00769).

## Conflicts of Interest

The authors declare no conflicts of interest.

## Supporting information

Supplementary Table 1.

## Data Availability

The data that support the findings of this study are available from the corresponding author upon reasonable request.
