# Supplementary material for: Diagnostic Performance of a Combined Rapid Antigen Test for Detecting SARS‐CoV‐2, Influenza Virus, and Respiratory Syncytial Virus in Symptomatic Patients in Tertiary Care
Source: J Med Virol. 2025 Jul 16;97(7):e70493. doi: 10.1002/jmv.70493 (PMC12265398; doi:10.1002/jmv.70493)
Supplement: Supplementary file 1 — Supplementary Table 1. [file JMV-97-e70493-s001.docx]

**Supplementary Table 1.** Percent agreement and Cohen’s kappa score for comparison of Xpert® Xpress SARS-CoV-2/Flu/RSV and the Alltest-SARS-CoV-2/IV-A+B/RSV in clinical respiratory swabs (n=100).

| **A** |  |  | Xpert® Xpress  SARS-CoV-2 | |  | |  |
| --- | --- | --- | --- | --- | --- | --- | --- |
|  |  |  | positive | negative | | Agreement | Kappa |
| Alltest-  SARS-CoV-2 | positive |  | 24 | 0 | | 84.0% | 0.64 |
|  | negative |  | 16 | 60 | |  |  |

| **B** |  |  | Xpert® Xpress  IV-A/B | |  | |  |
| --- | --- | --- | --- | --- | --- | --- | --- |
|  |  |  | positive | negative | | Agreement | Kappa |
| Alltest-  IV-A/B | positive |  | 19 | 0 | | 84.0% | 0.61 |
|  | negative |  | 16 | 65 | |  |  |

| **C** |  |  | Xpert® Xpress  RSV | |  | |  |
| --- | --- | --- | --- | --- | --- | --- | --- |
|  |  |  | positive | negative | | Agreement | Kappa |
| Alltest-  RSV | positive |  | 15 | 0 | | 90.0% | 0.69 |
|  | negative |  | 10 | 75 | |  |  |
